# High-Temperature Oxidation and Phase Stability of AlCrCoFeNi High Entropy Alloy: Insights from In Situ HT-XRD and Thermodynamic Calculations

**DOI:** 10.3390/ma17143579

**Published:** 2024-07-19

**Authors:** Muhammad Arshad, Saira Bano, Mohamed Amer, Vit Janik, Qamar Hayat, Mingwen Bai

**Affiliations:** 1 Centre for Manufacturing and Materials–Materials Science, Coventry University, Coventry CV1 5FB, UK; arshadm15@uni.coventry.ac.uk (M.A.); amerm5@uni.coventry.ac.uk (M.A.); ac6600@coventry.ac.uk (V.J.); qamar.hayat@warwick.ac.uk (Q.H.); 2 Department of Chemical Engineering, University of Engineering and Technology, Peshawar 25120, Pakistan; sairabano@uetpeshawar.edu.pk; 3 School of Mechanical Engineering, University of Leeds, Leeds LS2 9JT, UK

**Keywords:** phase stability, ThermoCalc, CALPHAD method, oxidation, AlCrCoFeNi HEA, in situ X-ray diffraction

## Abstract

The high-temperature oxidation behaviour and phase stability of equi-atomic high entropy AlCrCoFeNi alloy (HEA) were studied using in situ high-temperature X-ray diffraction (HTXRD) combined with ThermoCalc thermodynamic calculation. HTXRD analyses reveal the formation of B2, BCC, Sigma and FCC, phases at different temperatures, with significant phase transitions observed at intermediate temperatures from 600 °C–100 °C. ThermoCalc predicted phase diagram closely matched with in situ HTXRD findings highlighting minor differences in phase transformation temperature. ThermoCalc predictions of oxides provide insights into the formation of stable oxide phases, predominantly spinel-type oxides, at high *p*(O_2_), while a lower volume of halite was predicted, and minor increase observed with increasing temperature. The oxidation behaviour was strongly dependent on the environment, with the vacuum condition favouring the formation of a thin, Al_2_O_3_ protective layer, while in atmospheric conditions a thick, double-layered oxide scale of Al_2_O_3_ and Cr_2_O_3_ formed. The formation of oxide scale was determined by selective oxidation of Al and Cr, as further confirmed by EDX analysis. The formation of thick oxide in air environment resulted in a thick layer of Al-depleted FFC phase. This comprehensive study explains the high-temperature phase stability and time–temperature-dependent oxidation mechanisms of AlCrCoFeNi HEA. The interplay between surface phase transformation beneath oxide scale and oxides is also detailed herein, contributing to further development and optimisation of HEA for high temperature applications.

## 1. Introduction

At elevated temperatures, the movement of atoms and ions within materials increases due to the involvement of activation energy. Additionally, the concentration of vacancies, which facilitate atomic and ionic diffusion, rises exponentially with temperature [1]. Consequently, any changes that occur within alloys, both internally (phase change) and on the surface (oxidation/corrosion), which degrade their properties, are significantly accelerated at high temperatures. The design of high-temperature alloys aims to mitigate these deleterious changes. Oxidation, hot corrosion, creep, thermal fatigue, and microstructural evolution are modes of alloy degradation that can occur at high temperatures [2]. Environmental degradation modes like oxidation and hot corrosion are particularly challenging due to the inherent difficulties in controlling the service environment [3]. To counter these deteriorations at high temperatures, it is essential to either employ materials that naturally form protective layers or to apply such coatings to the alloy surfaces. These coatings form a barrier that stops further degradation, thereby maintaining the structural integrity of the material when subjected to extreme thermal challenges.

The creation of a stable, coherent, and protective oxide layer on high-temperature alloys is crucial for their longevity and functionality when subjected to extreme temperatures and corrosive atmospheres, thereby reducing the chances of pre-mature material failure [4,5]. In particular, the development of protective alumina scales, principally containing chromia Cr_2_O_3_ (Chromia) and Al_2_O_3_ (alumina), is a defining characteristic intended for the category of alumina-forming alloys. This group encompasses Fe-Cr-Als (Iron-based) [4], Ni-Cr-Als (Nickel-based) [6], Co-Cr-Als (Cobalt-based) [7], and NiAl (Nickel-aluminide) materials. The selection of these alloys is tactical for applications that operate at temperatures above 900 °C, where their capacity to form such scales guarantee their durability and prolonged service life.

Various beneficial properties of α-Al_2_O_3_ scales underpin their choice for improving the oxidation resistance of high-temperature alloys: (i) scale thickness: α-Al_2_O_3_ scales are characterised by their slow growth rates, which encourages a steady and controllable thickness growth over extended periods. (ii) Environmental Stability: The natural thermodynamic stability of α-Al_2_O_3_ guarantees that the scale remains intact and effective, even in aggressive environments, thereby extending the life span of the alloy. (iii) Inertness: The chemical passivity of α-Al_2_O_3_ scales acts as an impervious shield, preserving the alloy from oxidative damage and maintaining its structural integrity [4,5].

In materials research, the search for alloys that demonstrate excellent high-temperature performance remains a critical focus, driven by the demand for enhanced efficiency in high-temperature applications. High entropy alloys (HEAs), with their unique composition of various principal elements either in equal or in nearly equal amount, stand at the forefront of this quest [8]. These alloys possess combination of exceptional qualities, including inherently high melting points, formidable resistance to high-temperature deformation (creep), enhanced wear resistance, and a robustness in microstructural integrity [6]. These alloys commonly exhibit as single or multiphase metallic solid solutions, adopting diverse crystallographic arrangements such as FCC, BCC, and HCP structures [9,10,11].

Although HEAs exhibit impressive melting points and phase stability that align with the needs of demanding applications, their capability basically depends essentially on the development of a protective type oxide layer—a characteristic also key to the functionality of conventional Fe-based and Ni-based alloys [5]. Current studies on HEAs high-temperature phase stability and oxidation behaviour primarily focus on post-mortem analysis of isothermal oxidation products and phases formed after annealing. Research has focused on the CrFeCoNiX (X = Si, Mn, Al) [9,12,13] and CrMoTiAlY (Y = Ta, Nb, W) [14] alloys systems, with findings highlighting the beneficial effects of alloying elements like Cr [15] and Al [12] on oxidation resistance as well as the negative effect of elements such as Mn [12,16] and Cu [17] which can lead to the formation of unstable surface oxides prone to cracking and spallation. However, these studies are limited by their focus on characterising the oxide layer/phases after a fixed oxidation/annealing time, failing to capture the dynamic nature of the oxidation process and the underlying mechanisms at play. Despite the progress from these studies in understanding the oxidation behaviour and phase stability of HEAs, there remain notable gaps in the literature. Most oxidation studies have relied on post-mortem analysis of reaction products, which introduces uncertainty about the underlying physical mechanisms during the dynamic oxidation process. In situ techniques like high-temperature XRD (HTXRD) can provide important understandings into the real-time formation of oxides, phase transitions, and the temperature-dependent behaviour of HEAs under oxidative conditions.

In this study, we aim to track high-temperature thermal phase stability and oxidation behaviour of HEAs, with a specific focus on the AlCrCoFeNi alloy. This alloy is distinguished by its high-temperature strength and the pivotal role its high aluminium content can play in forming exclusive alumina scale. Previous research [18] into phase stability and oxidation behaviour of AlCrCoFeNi-based HEAs is isothermal (i.e., analyses are based on left-over product), with practically no studies addressing its in situ phase formation and oxidation behaviour. Our research utilises in situ X-ray diffraction (HTXRD) to accurately predict the temperature of phase transformation and oxidation initiation with increasing temperature and time. This experimental approach is reinforced by thermodynamic equilibrium calculations using ThermoCalc Software 2024a (Stockholm, Sweden) with the latest TCHEA7 database. These calculations predict stable phases and oxidation behaviour under experimental conditions. This allows for a detailed comparative analysis of the results. Additionally, by integrating in situ XRD with Thermo-Calc predictions, this study provides insights into the time–temperature-dependent oxidation mechanism and phase transformations, offering a clearer understanding of the stable phases present under different conditions.

## 2. Experimental Details

### 2.1. Sample Preparation

The manufacturing of materials involved using high-purity elements (greater than 99.8% purity, sourced from Alfa Aesar Thermo Fisher Scientific, Waltham, MA, USA) and employing the Arc-melting technique. The specifics of the sample preparation through the arc melting process have been detailed in our previous study on CrCoFeMnNi [19]. The resulting cast alloy was formed into ingot. The ingot was then sectioned into 1.6-mm-thick slices using Electrical Discharge Machining (Microtec EDM, Essex, UK), and further processed into 20 × 20 mm square samples for further experimental analysis. Each sample was ground and polished using the standardised Struers method, with 1.0 μm of diamond paste used in the final polishing step to achieve the desired surface finish.

### 2.2. Microstructure Analysis

As-casted and heat-treated (sample used for in situ HTXTD at 1100 °C) samples microstructure examination was conducted employing SEM (scanning electron microscopy, Car ZEISS, Oberkochen, Germany) having energy-dispersive X-ray spectrometer (EDS, Car ZEISS, Oberkochen, Germany) on a Carl ZEISS Gemini SEM: (Zeiss Sigma 500 VP, Oberkochen, Germany). SEM was used in BSE mode for phase contrast imaging of heat-treated and as-cast HEA specimen. In addition, qualitative and quantitative elemental analysis was carried out using SEM enabled energy-dispersive X-ray spectroscopy (EDX, Car ZEISS, Oberkochen, Germany) technique, providing chemical composition data and distribution of elements in the alloy sample as well as within the oxide layers. Furthermore, cross-sectionals were prepared, with each sample’s oxide scale protected from spallation by embedding it in a ConduFast epoxy resin. Metallographic preparation corresponded the methodology used for the samples, and cross-sections were examined using SEM+EDX techniques after carbon coating to mitigate sample charging.

### 2.3. Diffraction Analysis

Structural characterisation of the substrate AlCrCoFeNi HEA and oxides was done using XRD (Bruker, Karlsruhe, Germany) using standard Bragg–Brentano geometry. Analysis of the results utilised EVA combined with Xpert HighScore Plus 3.05 (Bruker, Karlsruhe, Germany) using ICDD PDF2 data and crystallography open database combined with the published literature on isothermal oxidation of the alloy in various temperatures. The following sections provide details of the two non-ambient conditions for in situ HTXRD.

#### In Situ XRD

The crystal structure and oxidation behaviour of alloy at high temperature was analysed using high temperature X-ray diffractometer (HTXRD Bruker D8-Advance, (Bruker, Karlsruhe, Germany)) with Cu K_α1+2_ radiation operating at 40 kV/40 mA. The specimen for analysis was placed in hot stage (Anton Paar Domed Hot Stage, DHS1100, Anton Paar, Graz, Austria) equipped with graphite dome for creating and maintain experimental conditions (i.e., temperature, oxidative and vacuum environment). This state-of-the-art equipment enables precise control over the sample environment during non-ambient experiments. Two distinct experimental configurations were carefully designed as detailed in the following section for data collection process of the non-ambient conditions.

First, the phase stability and oxidation behaviour of as-cast AlCrCoFeNi HEA was conducted in lower oxygen partial pressure (*p*(O_2_)) (also referred as vacuum condition) using in situ HTXRD. A high vacuum level of approximately 4.2 × 10^−6^ atm was maintained in the hot stage using a turbo pump throughout the experiment. However, our setup does not measure oxygen partial pressure directly; instead, we estimate it from the overall vacuum level. Acknowledging this limitation, we plan to explore the integration of a zirconia oxygen probe or similar technology with our equipment manufacturer for more precise measurements. Second, the oxidation behaviour of AlCrCoFeNi HEA was investigated in air environment using in situ XRD, enabling real-time analysis of oxide formation and growth during high-temperature exposure. For oxidation in air environment, vacuum pump was kept off and atmospheric air conditions were maintained during heating and scanning. Under both experimental conditions, XRD patterns were recorded from 50 °C to 1100 °C after each 50 °C rise in temperature while the sample was held for 5 min at each temperature setpoint before XRD scan. In both cases, the heating rate was kept constant at 10 °C/min from one setpoint to the next. Scanning was performed in 2θ range of 30–80° with a scanning speed of 0.102 s per step. In both cases, a polished sample was positioned on an AlNi (aluminium nitride) plate in the hot stage dome for analysis.

### 2.4. Thermodynamic Calculations

The CALPHAD approach has been commonly used for the study of phase equilibria of multi-component system [20]. This powerful technique combines thermodynamic principles, experimental data, and computational modelling to predict and analyse the phase stability, thermodynamic properties, and phase transformations of materials across a wide range of compositions and temperatures. In this work, thermodynamic calculations facilitating the determination of equilibrium phases in AlCrCoFeNi alloys were performed using Thermo-Calc software with the TCHEA7 database for high entropy alloys. The phases predicted by ThermoCalc were visualised in one step phase diagram in relation to the phase fraction of the alloy with temperature under conditions of extended annealing time to permit complete atomic diffusion of elements to reorder themselves and form the most thermodynamically stable configurations with optimum Gibbs free energy.

Thermodynamic calculations were carried out for the formation of oxide scale on the AlCrCoFeNi alloy at extreme temperatures, considering oxygen partial pressures *p*(O_2_) at different temperature. Database for Ni-Superalloy (TCNi11) was used for equilibrium computations. Thermo-Calc calculations proved helpful for studying selective oxidation properties in high-temperature alloys, especially in multi-component alloys. The calculations were performed under equilibrium conditions, corresponding to the experimental conditions in the in situ HTXRD oxidation test used in this study. The oxygen effective (*p*O_2_) was treated as a variable, represented by chemical activity (a), varying from 1.0 × 10^−14^ atm to 1.0 atm. Table 1 shows the equilibrium conditional variables for determining the oxides phase composition formed on the alloy. It is important to note that thermodynamic calculations, that focus on equilibrium conditions, do consider kinetic effects such as diffusion, nucleation, and reactions on surface, which affect the rate of oxidation and formation of oxide layers in high-temperature environments. Additionally, the accuracy of these calculations is contingent on the quality of databases, relying on experimental data for model parameter optimisation and validation. Variations in data quality and reliability, particularly for less studied alloy systems or at high temperatures, can lead to discrepancies between calculated and experimental results.

## 3. Results

### 3.1. Composition and Microstructure of AlCrCoFeNi HEA

**Figure 1:**
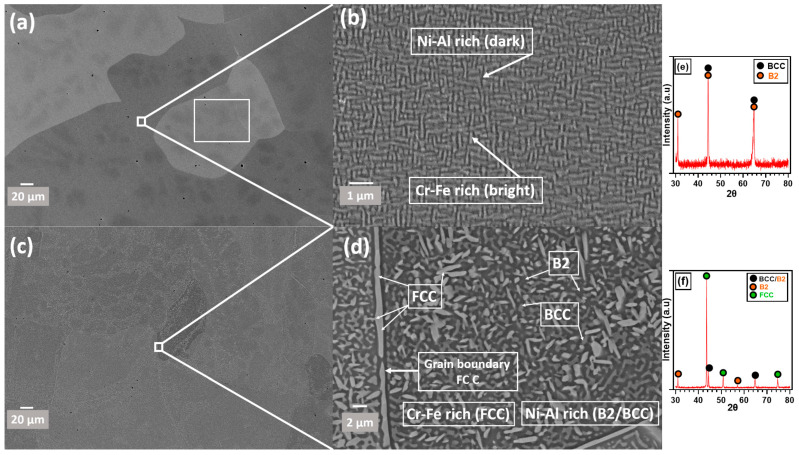
SEM images of an AlCrCoFeNi HEA: (**a**) as-cast microstructure with rectangular scan area for EDX analysis, (**b**) magnified view of the as-cast maze-like structure of bright and black combination (**c**) sample heat-treated (i.e., HTXRD sample heated up to 1100 °C) (**d**) magnified image of heat treated with ultra fine micro platelet microstructure, (**e**) XRD scan of virgin sample (**a**), (**f**) XRD scan at RT of heat treated (i.e., HTXRD sample heated up to 1100 °C).

Figure 1 illustrates the microstructure of AlCrCoFeNi HEA as-cast and heat-treated (sample after HTXRD 1100 °C) sample specimen. The as-cast sample manufactured through arc melting process is a mixture of two phases BCC with a space group Im-3m (≈79%) and BCC_B2 (≈21%) with a space group (Pm-3m) as shown by XRD scan in Figure 1e. The sample employed for in situ XRD analysis, referred to as the heat-treated sample, underwent a transformation of its dual-phase structure (BCC and B2) and predominantly retained its FCC phase upon cooling from 1100 °C to room temperature. The XRD pattern at room temperature of this heat-treated sample is shown in the Figure 1f.

The AlCoCrFeNi high-entropy alloy (HEA) consists of two nanostructured phases uniformly distributed within each grain, forming a maze-like pattern (Figure 1a,b). This microstructure is a result of the spinodal decomposition mechanism [21]. Figure 1c,d show the heat-treated BSE mode images of the AlCrCoFeNi HEA. During in situ HTXRD, the disordered body-centred cubic (BCC) phase decomposes into the sigma (σ) phase and face-centred cubic (FCC) phase above 600 °C (see XTXRD in Low p(O_2_) conditions). Further heating above 950 °C leads to the decomposition of the σ phase into FCC and BCC phases, as shown in Figure 1c,d. Further to confirm the EDX chemical analysis of the as-cast HEA sample is slightly different from the prepared mixture (equal atomic %age) for alloy manufacturing and is shown in Table 2. Aluminium at.% is decreased as reported in some work [22] while at% of the remaining elements is more than that of nominal composition.

### 3.2. Thermodynamic Analysis of AlCrCoFeNi High Entropy Alloy

Studies have attempted to calculate phase constitution in high-entropy alloys in relation to composition using thermodynamic models, particularly the CALPHAD method [23,24]. These models assume that the equilibrium state corresponds to the minimum Gibbs free energy of the system, with most stable phases having the minimal Gibbs free energies. The simulations were compared with experimental results to validate their effectiveness.

**Figure 2:**
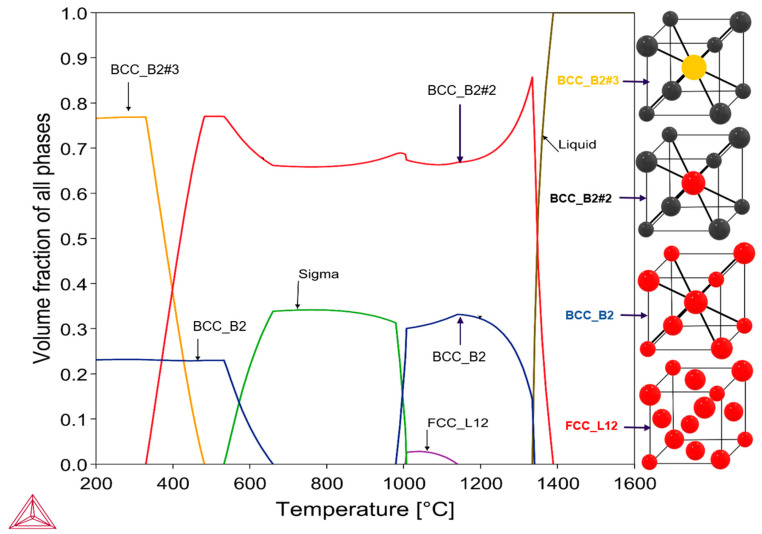
Thermodynamic Calculations of phase diagram for equi-atomic AlCrCoFeNi HEA performed using CALPHAD method and TCHEA7 database in ThermoCalc. The two main phases found were BCC (i.e., BCC_B2), and B2 and its variants (i.e., BCC_B2#2, BCC_B2#3), minor FCC, and sigma.

**Figure 3:**
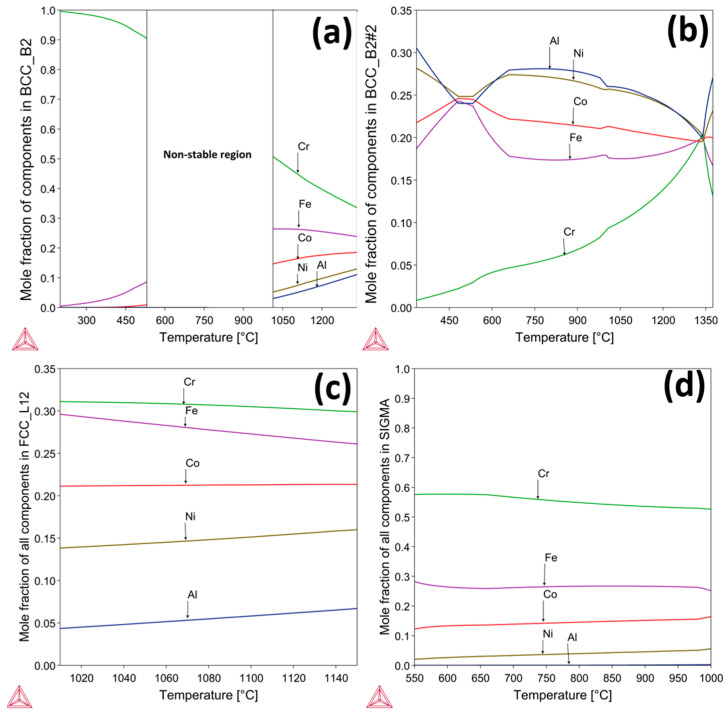
Shows elemental composition of predicted phases (**a**) mole-fraction of all components in the BCC phase over a wide temperature range, with a non-stable region indicated, (**b**) mole fraction of all components in the B2 phase from 450–1350 °C, (**c**) mole fraction of all components in the FCC_L12 phase from 1020–1140 °C, and (**d**) mole fraction of all components in the SIGMA phase from 550–1000 °C.

Figure 2 shows the one-step phase diagram of AlCrCoFeNi HEA calculated by ThermoCalc. Based on equi-atomic composition of AlCrCoFeNi HEA, calculations were performed to predict the stable phases over a temperature range of 200–1600 °C. The equilibrium phase composition is complex mixture of phases. At low temperatures (below 300 °C), a dual phase structure exists, a disordered BCC (i.e., BCC_B2) and an ordered BCC (BCC_B2#2 or variant BCC_B2#3). The elemental phase constitution of the calculated phases is summarised in Figure 3. As the temperature rises, the comparative amount of the B2 (i.e., BCC_B2#3) and BCC (i.e., BCC_B2) phases reduces and is fully converted when temperature approaches 650 °C. This transformation leads to the formation of the B2#2 phase a variant of B2 starting at 300 °C and a new sigma phase starting at 550 °C. The sigma phase is CrFe-rich with small amounts of Co and Ni (see Figure 3d). It is formed at the cost of BCC phase (Cr-Fe-rich) together with some Co and Fe from the B2#2 phase (seen as a decrease in the amount of Fe and Co around 550 °C in Figure 3b for the B2#2 phase). At the intermediate temperature between 675 and 975 °C, the alloy contains two phases: the AlNi-rich B2#2 phase and the CrFe-rich sigma phase. The sigma phase becomes unstable at higher temperatures and is completely reinstated by the new BCC (i.e., BCC_B2) phase at around 1000 °C. Above this temperature, the alloy consists of two phases: the B2#2 phase and the new BCC phase. There is also a small amount of an FCC_L12 phase that is stable between 1000 °C and 1100 °C.

### 3.3. AlCrCoFeNi HEA In Situ High Temperature XRD

In situ thermal observations of materials using high-temperature XRD provide critical data on temperature-dependent phase transformations, the formation of new phases, and the start and finish temperatures of participating phases within an alloy system [25]. To reveal real time oxide formation along with temperature at which an oxide form in situ HTXRD is having critical role to demonstrate its capability in revealing underlaying mechanisms in diverse environmental conditions.

#### 3.3.1. HTXRD in Low *p*(O_2_) Conditions

**Figure 4:**
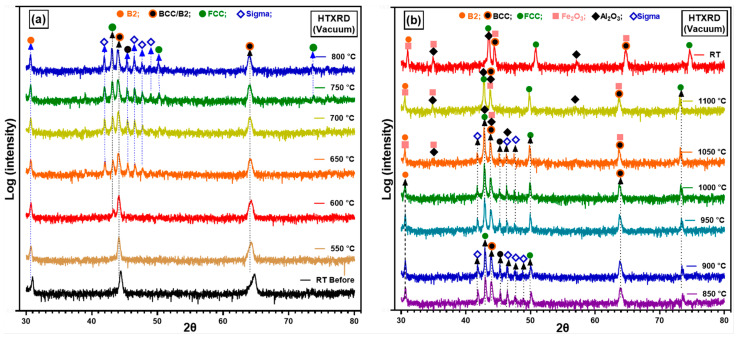
The figure shows in situ XRD scans of an AlCrCoFeNi alloy performed in a vacuum environment from RT to 1100 °C. Image (**a**) shows the evolution of the diffraction peaks with increasing temperature, while image (**b**) shows data of same sample for high temperatures with each peak indexed for phase identification, revealing the presence of BCC, B2, and formation of FCC, Sigma, and oxide phases. The diffractograms are vertically shifted for clarity.

Low oxygen partial pressure conditions (*p*O_2_ = 8.82 × 10^−7^ atm) during in situ HTXRD were used to investigate phase structure of the as-cast alloy. Further, to study the effect of low *p*(O_2_) on the oxidation of AlCrCoFeNi HEA during the in situ HTXRD. The results of phase transition of the substrate alloy (AlCrCoFeNi HEA) and oxidation at low *p*(O_2_) during in situ HTXRD, detailed in Figure 4a,b, explain the phase formation and transformations of the AlCrCoFeNi HEA from room temperature (RT) up to 1100 °C, followed by subsequent cooling to room temperature. Diffractograms for temperatures up to 500 °C are skipped, as they primarily exhibit peak shifts due to the expansion of lattice parameters with increasing temperature.

As seen in Figure 1a, the XRD scan at 600 °C shows the emergence of a new peak at a 2θ value of 43.25° which indicates the initiation of the FCC phase, shown by green dots. As the temperature rises to 650 °C, additional peaks were observed at 2θ values of 45.32°, 46.41°, and 47.55° and are identified as the sigma phase, denoted by blue hollow diamonds. The peaks associated with the sigma phase increased in intensity up to 850 °C. Figure 4b shows the scans up to the RT after cooling, the scan at 850 °C shows the intensity of the sigma phase peaks begins to decline and completely disappeared in a scan at 1050 °C. At the same time observed the formation of Fe_2_O_3_ (marked by pink squares) and Al_2_O_3_ (marked by black diamonds) starting at 900 °C. By 1100 °C, XRD scans analysis reveals the presence of Fe_2_O_3_ and Al_2_O_3_ oxides along with the FCC phase and the alloy initial phases.

After the sample was cooled down to room temperature, a significant change in phase types and contribution is evident. The alloy primarily exhibits the FCC and B2 phases, with the absence of the sigma phase and a notable reduction in the BCC phase. This change suggests that certain high-temperature phases, particularly the sigma phase, either decompose or transform upon cooling, leading to a more stable configuration of B2/BCC and FCC phases at RT.

**Figure 5:**
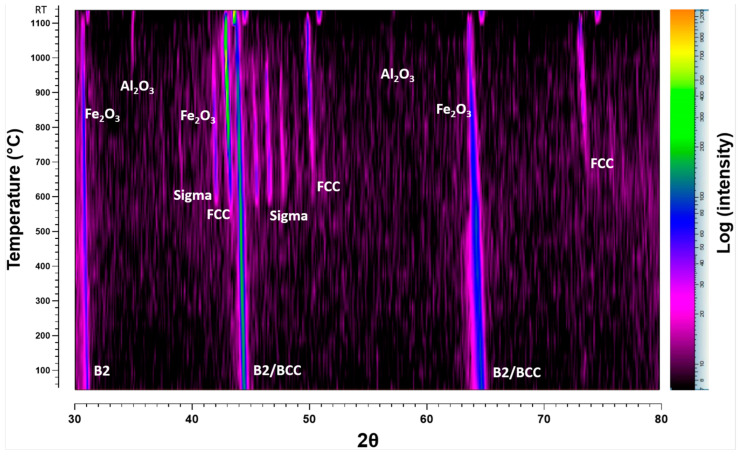
Shows AlCrCoFeNi HEA densitometric view recorded during in situ HTXRD in vacuum condition while heating from RT to 1100 °C.

High-temperature X-ray diffraction (HTXRD) data in the form of densitometric view for AlCrCoFeNi HEA is shown in Figure 5, illustrating the evolution of diffraction intensity across various crystallographic planes from room temperature to 1100 °C. Initially, the dominant phase is B2/BCC phases appearing as the room temperature phases. The FCC phase emerges at around 550 °C, followed by the sigma phase at 600 °C. Significant oxide formation is observed at 800 °C with peaks for Fe_2_O_3_ and Al_2_O_3_, which persist and intensify at higher temperatures, indicating robust oxidation processes. This detailed analysis underscores the alloy’s structural changes and phase transformations under thermal stress.

#### 3.3.2. In Situ XRD in Atmospheric Conditions

**Figure 6:**
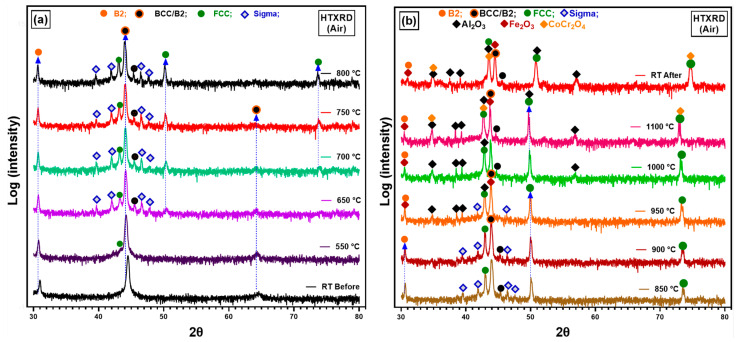
The figure shows in situ XRD scans of an AlCrCoFeNi alloy completed in air environment up to 1100 °C. Image (**a**) shows the evolution of the diffraction peaks with increasing temperature, while image (**b**) shows the same data for high temperature each peak is indexed showing the corresponding phase.

In situ HTXRD was carried out in atmospheric conditions (air) so that the alloy should have sufficient oxygen availability and to study the oxidation process of AlCrCoFeNi. Results of diffraction patterns of in situ HTXRD study on arc-melted alloy are shown in Figure 6. The scans in the temperature range were conducted to monitor the phase transformation and especially oxidation process under atmospheric conditions when there is no *p*(O_2_) drop on the sample or on the formed oxide surface. As-cast room temperature XRD scan of the alloy shows body-centred cubic (BCC) and B2 phases were exposed to a controlled heating and XRD patterns recorded at 50 °C increments and was gradually heated to track the variations in the alloy’s phase structure and oxides initiation. From RT to 500 °C, XRD scans are not included due to no change in scans revealing stability of phases in this temperature range. This persistence of phases suggests that the alloy maintains its primary structure up to this temperature range. The first noticeable change was observed at 550 °C as earlier noticed in vacuum conditions, with a new peak which was later confirmed the formation of face-centred cubic (FCC) phase. This indicates the inception of phase transformation, representing the transition from primary BCC and B2 structures to a new FCC phase. At 650 °C, a new phase began to appear, marked by blue hollow diamond the σ (Sigma) phase alongside BCC/B2 and FCC. With further increase in temperature at 750 °C, peak at 2θ value of 64.2 belonging to B2/BCC phases diminished, indicating a reduction in the B2/BCC phase as FCC and sigma phases grew. These patterns clearly indicate that the as-cast AlCrCoFeNi HEA is not stable at intermediate temperature. The σ phase continued to develop with increasing temperature, reaching a peak in intensity at around 800 °C, and diminishes when temperature reached 950 °C. At 950 °C, sigma peaks had almost disappeared, and new peaks appeared that were expected to belong to oxides of Al_2_O_3_, Fe_2_O_3_. Increases in the new phase intensities belonging to oxides continued showing the continuous oxide formation. The oxidation process became pronounced at 1000 °C and continued through to 1100 °C, as evidenced by the prominence of oxide peaks in the XRD patterns. The higher temperatures favoured the formation and growth of complex oxide structures. After the sample cooled to room temperature, the final XRD pattern (‘RT After’) showed the residual phases. The BCC, B2, and FCC remained as the primary phases of AlCrCoFeNi HEA. Oxide phases such as Al_2_O_3_, Fe_2_O_3_, and CoCr_2_O_4_ were also retained, demonstrating the stability of these oxides post-high-temperature exposure.

**Figure 7:**
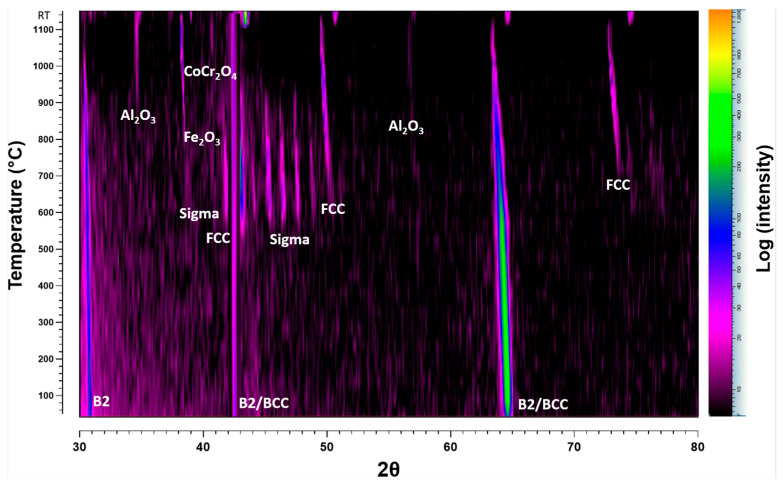
Shows the densitometric view of AlCrCoFeNi HEA in air conditions.

Figure 7 shows densitometric view of in situ XRD conducted in an air atmosphere and illustrates the evolution of diffraction intensity for the AlCrCoFeNi HEA as temperature increases from RT to 1100 °C. It provides a clear view of phases, phase evolution and transformation. The FCC phase emerges at approximately 550 °C, followed by the sigma phase around 600 °C. Significant oxidation occurs at 900 °C with the formation of Fe_2_O_3_ and Al_2_O_3_, which intensify at higher temperatures. At 1100 °C, CoCr_2_O_4_ is also observed, indicating complex oxidation behaviour.

### 3.4. Oxide Scales Surface Analysis

#### 3.4.1. Surface Oxides Formed in Air Conditions

**Figure 8:**
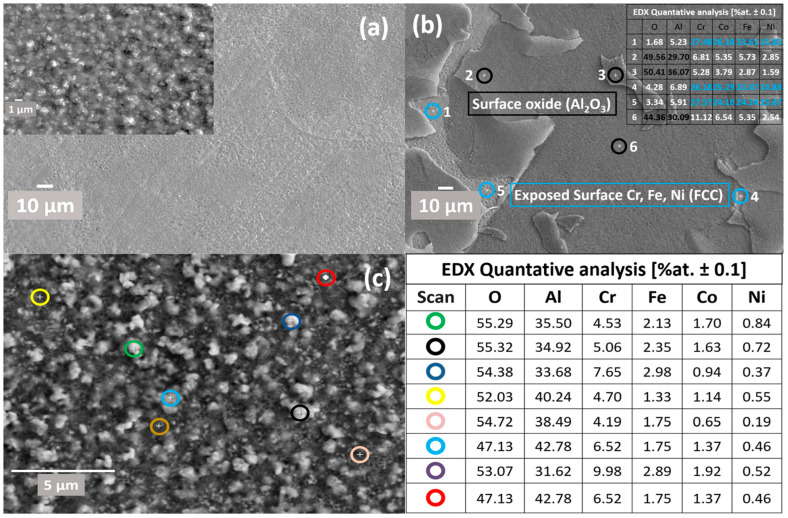
SEM analysis of alloy surface oxidised in air. (**a**) Low-magnification image revealing the surface morphology with a high-magnification inset. (**b**) SEM image of the oxidised surface exhibiting oxide spallation, with the corresponding table presenting EDX point scan results of oxide and exposed surface. (**c**) EDX image showing EDX point scans, accompanied by a table summarising the elemental composition (at%), indicating the development of (Al_2_O_3_) an Al-rich oxide layer.

Figure 8 shows a comprehensive characterisation of the oxide scale formed on an equi-atomic AlCrCoFeNi High Entropy Alloy (HEA) subjected to high-temperature oxidation during in situ XRD. Figure 8 consists of three scanning electron microscope (SEM) images, labelled (a), (b), and (c), and an elemental composition, collectively providing a detailed analysis of the oxide surface morphology, topography, and chemical composition. Figure 8a exhibits the overall surface morphology and topography of the oxidised HEA at two different magnifications. The main image reveals a rugged surface, while the higher magnification inset displays a noodle-like structure. This morphology is characteristic of thermally grown oxide scales on high-temperature alloys, resulting from the complex and uneven formation of oxide layers under dynamic and reactive oxidation conditions [26].

Figure 8b shows where the oxide layer spallation has occurred, exposing the underlying alloy surface. Oxide scale spallation in AlCrCoFeNi HEA has previously been reported [27,28] to be due to the high cooling rate, which creates a more aggressive impact due to CTE (Coefficient of Thermal Expansion) mismatch. This phenomenon of spallation following high-temperature oxidation, as observed in our alloy, aligns with findings reported by Dąbrowa et al. In their study [27] on the AlCoCrFeNi alloy subjected to 100 h of oxidation, they noted spallation of the oxide layer, which occurred primarily during the cooling phase. This observation was correlated with TGA results indicating mass loss during cooling, attributed to the differential thermal expansion coefficients (CTEs) between the oxide layer and the underlying alloy. This disparity in CTEs is recognized as a fundamental cause of spallation due to induced mechanical stresses during thermal contraction.

Chemical composition analysis was conducted at six locations as shown in Figure 8b: three on the oxide surface (marked with black circles) and three on the exposed surface (marked with blue circles). The oxide surface is enriched in Al and O, confirming the predominant formation of alumina (Al_2_O_3_), its presence is also verified by in situ X-ray diffraction (XRD) (see Figure 6). Alumina layers are renowned for their protective properties, enhancing the alloy’s oxidation resistance at elevated temperatures. The exposed surface exhibits a composition rich in Cr, Fe, and Ni, as Co distribution in these alloys is uniform in different phases (B2/BCC, FCC). Figure 8c provides a higher magnification view of the oxide layer surface morphology; it shows details of the oxide scale’s granularity and texture. EDX was used for chemical composition analysis at eight locations. The analysis on this surface reveals a composition primarily dominated by Al, followed by Cr. This composition reflects the protective nature of the formed oxides, mainly alumina with contributions from chromium oxides, which are essential for the alloy’s performance at high temperature. Chemical analysis of eight different points of the oxide surface is shown in the accompanying table as elemental atomic percentages (at.%), highlighting the significant presence of Al and Cr.

#### 3.4.2. Surface Oxidation in Vacuum Conditions

**Figure 9:**
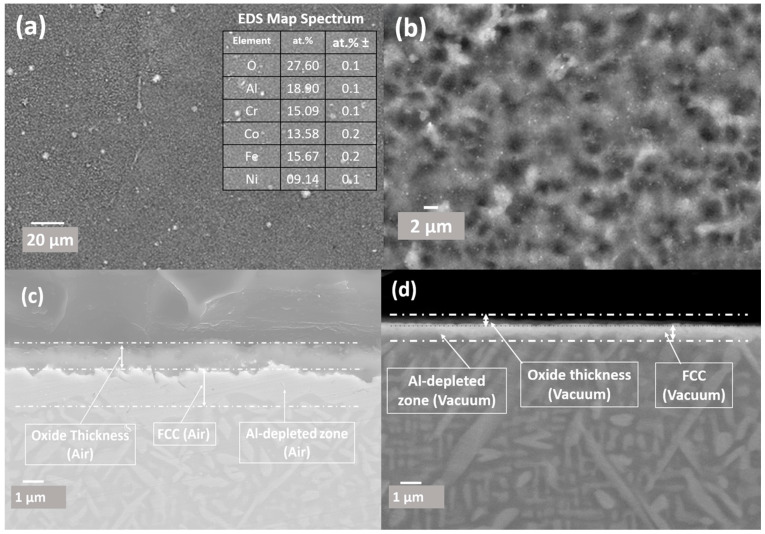
(**a**) Sample surface oxide in vacuum, (**b**) magnified view of the surface oxide morphology, (**c**,**d**) comparison between the oxides of the AlCrCoFeNi HEA under air and vacuum conditions.

Surface and cross-sectional analysis of AlCrCoFeNi high-entropy alloy (HEA) formed during in situ HTXRD under vacuum conditions is shown in Figure 9. XPS is indeed a useful technique for this kind of analysis. It provides a detailed understanding of surface chemistry and elemental oxidation states, which is critical due to its sensitivity to the outermost layers of the material, about 10 nm deep. Recognising its potential to enhance our findings, we plan to incorporate XPS in our future work to gain deeper insights into the oxidation mechanisms. The surface morphology revealed in Figure 9a shows a relatively smooth surface with fine-grained texture, suggesting that the oxide layer is thin and uniformly distributed. The associated table provides elemental compositional data of the image from EDX analysis map, showing the atomic percentages of elements. The high oxygen content confirms the formation of an oxide layer during the HTXRD process. The significant presence of aluminium, chromium, and iron suggests the formation of stable oxides such as alumina (Al_2_O_3_), chromium oxide (Cr_2_O_3_), and iron oxide (Fe_2_O_3_), which are known for their protective properties. The relatively high aluminium content indicates a dominant formation of alumina, contributing to the stability and protection of the oxide layer.

Figure 9b reveals a more detailed view of the surface oxide morphology, the texture appears more granular at high magnification. The granular appearance suggests the existence of a thin, continuous layer of oxide composed of fine crystallites. The overall uniformity and fine-grained texture of the oxide layer indicate a stable, protective oxide film that has developed under controlled low-oxygen conditions. The two images (Figure 9c,d) illustrate AlCrCoFeNi oxidation behaviour under different environmental conditions. Figure 9c shows the HEA oxidised in air, revealing a uniform and relatively thick surface oxide layer, with a noticeable formation of the FCC phase beneath this oxide layer. This indicates that the high *p*(O_2_) in the air promoted the growth of oxide layer, providing a protective barrier but also resulting in significant phase transformation beneath the oxide layer. In contrast, Figure 9d shows the HEA oxidised in vacuum conditions, characterised by low *p*(O_2_). Here, a much thinner oxide layer is observed, along with a thin layer of FCC phase underneath the oxide layer. The reduced oxygen availability in vacuum conditions limits the oxidation rate, while thin FCC layer indicates minimal phase transformation, as the low oxygen environment is less conducive to significant structural changes within the alloy.

### 3.5. Cross-Sectional Analysis of the Oxide Scales

#### 3.5.1. Oxides Cross-Sectional Analysis Formed in Vacuum

**Figure 10:**
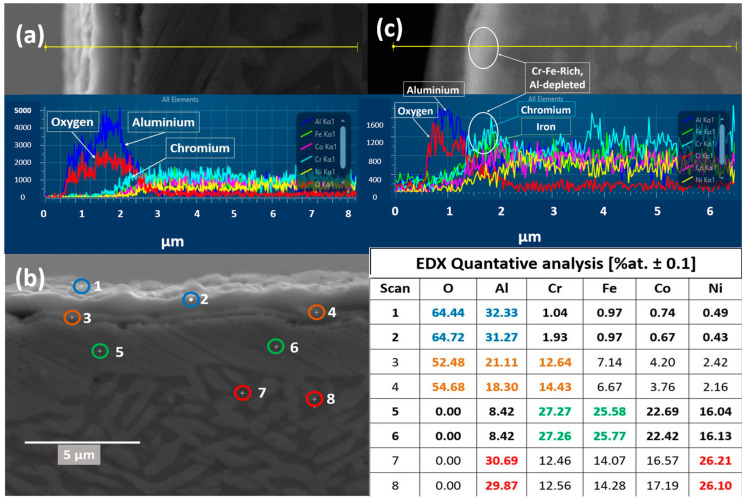
(**a**,**c**) EDX line scan showing elemental distribution of elements in oxide layer and alloy of samples oxidised in air and vacuum, respectively. (**b**) EDX image with EDX point scan locations. The table lists the elemental composition of the point scan analysis.

Figure 10c represents cross-sectional EDX lines scan analysis of the oxide layer formed on equi-atomic AlCrCoFeNi HEA in vacuum conditions during in situ XRD. From the line scan analysis in Figure 10c, it is evident that the external oxide layer is largely developed by Al forming Al_2_O_3_ along with a Cr_2_O_3_ internal subscale followed by Fe forming Fe_2_O_3_. Below, the oxide scale bright phase indicates the phase formed of elements of high atomic weights. As observed, EDX line scan analysis provides thickness of bright phase formed beneath the oxide scale indicating the consumption of Al from the alloy primary phases to form oxide and the corresponding phase transformation.

#### 3.5.2. Cross-Sectional Analysis of Oxide Formed in Air

Figure 10 shows a detailed EDX analysis conducted on the oxide sample cross-section to investigate the multi-layered oxide scale formed on AlCrCoFeNi HEA sample oxidised during in situ XRD. Figure 10a shows an EDX line scan analysis of the oxide layer formed in air. The line scan reveals a double-layered oxide structure primarily composed of two alloying elements, Al, and Cr, which are widely used in alloys to create protective surface oxide scale, preventing further degradation. The Al counts indicate that the top thick oxide layer consists of Al likely forming Al_2_O_3_, while a thin layer of scale beneath is composed of Cr suggesting the formation of Cr_2_O_3_. This shows that the oxide scale primarily consists of Al_2_O_3_ and Cr_2_O_3_. However, the presence of a spinel phase, likely CoCr_2_O_4_ along with Fe_2_O_3_, was also identified during in situ XRD at room temperature after cooling. Figure 10b shows EDX point scan analysis at locations of interest on the formed oxide and the underlying alloy. The chemical composition of all the points is provided in the adjacent table of Figure 10b. The chemical analysis was examined at two points (marked as 1 and 2) on the top layer and found to be exclusively alumina (Al_2_O_3_). EDX point analysis at two locations (3 and 4) on the bottom layer shows a mixed composition of Al, Cr, Fe, and Co, with Cr being the major element in this oxide layer after Al. The composition of the underlying alloy was also checked at four different locations. Scans on the bright phase (points 5 and 6) showed that the bright phase is formed by Cr, Fe, and Co, while the dark matrix phase (points 7 and 8) is formed mainly by Al and Ni. Additionally, the formation of a B2-denuded zone was observed below the oxide layer across the sample, suggests alterations in the microstructural phase distribution due to thermal treatment and oxidation process.

**Figure 11:**
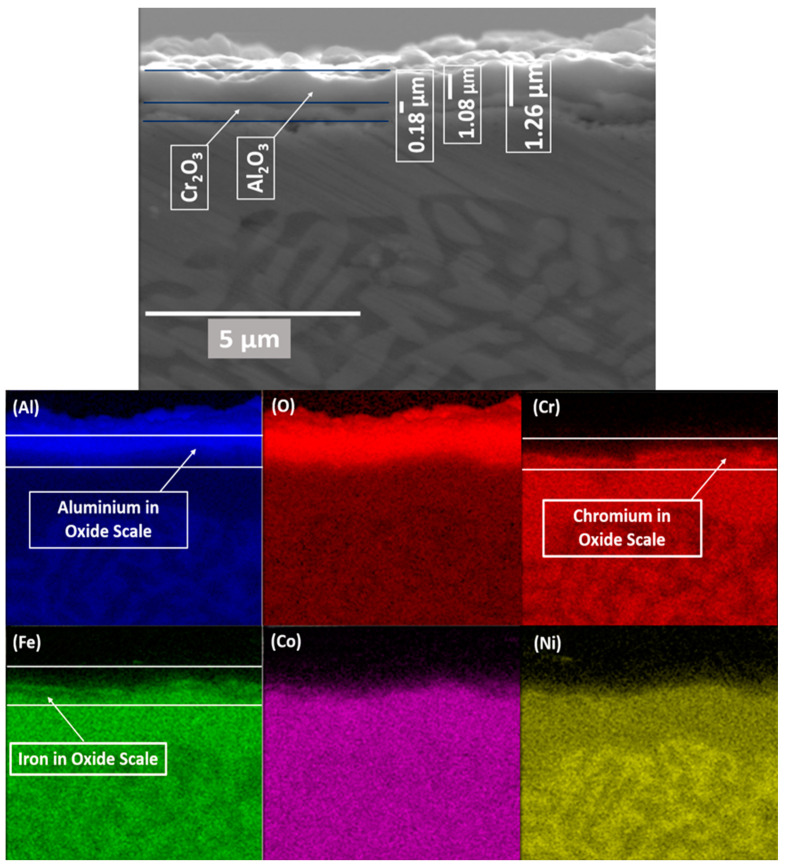
EDX map scan analysis of the oxide layer formed in air environment, double layer oxide scale Al_2_O_3_ and Cr_2_O_3_ thickness is shown in the image. Elemental map concentration shows high amount of Cr, Al, and Fe in the oxides formed.

Figure 11 illustrates EDX map scan analysis of cross-section of an oxidised sample to provide a visual representation of elemental distribution. Scan area image in Figure 11 exhibits EDX image of the cross-sectional view, used for map analysis. Scan area image in Figure 11 is labelled showing the multilayer oxide structure with a total thickness of 1.25 micrometres. This total thickness comprises primarily of an alumina layer measuring 1.08 micrometres, and the remaining thickness is from thin chromia layer.

The accompanying elemental distribution maps provide a graphic representation of the concentration and location of various elements within the oxide layers and the underlying alloy. The aluminium (Al) map shows a dense alumina layer at the surface, indicating a significant consumption of aluminium from the substrate to form this protective layer, resulting in an aluminium-depleted region just beneath the oxide layer. Elemental maps of both Fe and (Cr) reveals a thin but dense layers elemental concentration at the top, confirming the presence of oxide from Cr and Fe. Cobalt (Co) shows an even distribution within the alloy substrate but is notably absent in the oxide layers. Finally, the nickel (Ni) map displays a dim presence in the bright regions, indicating minimal presence in bright regions of substrate alloy. This distribution of elements underscores the selective oxidation behaviour of different alloying elements in HEAs, where specific elements such as aluminium and chromium play a dominant role in forming protective oxide [29].

## 4. Discussion

### 4.1. Phase Stability and Microstructural Evaluation in AlCrCoFeNi HEA

The findings from ThermoCalc for the AlCrCoFeNi high-entropy alloy (HEA) are generally consistent with in situ HTXRD phase stability investigations. ThermoCalc predicts the phase diagram for AlCrCoFeNi HEA, as shown in Figure 2. Below 300 °C, ThermoCalc estimates a mixture of BCC and B2 phases. In multi-component compounds, mixing appears in sub-lattices. The CALPHAD method uses IMA (ideal mixing assumption) in each sub-lattice followed by addition. This leads to an over-estimation and an expansion of the phase field. For instance, (see Figure 2) B2 of high-volume fraction (≈80%) is predicted in AlCrCoFeNi, in which experimentally a BCC exists in large amount (≈40%) [30]. For such phases, an enhanced method is to use IMA with stoichiometric weighting for each sub-lattice [31].

Composition of ThermoCalc predicted phases are summarised in Figure 3. The onset of the B2#2 phase above 300 °C, is an ordered variant of B2 and becomes a three-phase region (B2/BCC/B2#2), while in situ XRD indicates the occurrence of BCC and B2 phases. ThermoCalc predicts B2 and B2#2 as distinct ordered states, XRD typically only identify a single B2 phase because it lacks the resolution to detect the difference in atomic ordering [32]. The B2#2 phase formation is associated with the reordering of atoms within the B2 structure. The TCHEA7 database describes the variants of the B2 phase using a #n suffix, where n is an integer, to differentiate phases and is based on site occupancy.

As illustrated in Figure 2, phases volume fraction changes with temperature. Between 450 °C and 670 °C, ThermoCalc predicts that the initial BCC and B2 phases completely transform into B2#2 and sigma (σ) phases. Correspondingly, in situ XRD scans under vacuum conditions detect these changes with the beginning of new phase peaks at 550 °C and 650 °C, indicating the formation of FCC and sigma phases, Rogachev et al., (2021) also reports the formation of sigma phase in his work [33] during studying evolution of phases in AlCrCoFeNi HEA. The formation of these phases is in line with ThermoCalc predictions. However, the FCC phase was detected at a lower temperature (i.e., 550 °C) during in situ XRD, as opposed to the ThermoCalc prediction of over 1000 °C. This divergence is attributed to the non-equilibrium conditions of the experiment, where rapid heating or cooling rates lead to the formation of phases at temperatures different from those predicted under equilibrium conditions used by ThermoCalc [34].

In practical circumstances, the nucleation and growth of phases can occur at lower temperatures due to faster kinetics facilitated by defects or impurities in the material [33]. The observed differences between predicted and experimental phases in the AlCrCoFeNi HEA are primarily due to kinetic factors [34], non-equilibrium conditions during high-temperature XRD (HTXRD) [35], inaccuracies in thermodynamic databases [36], and microstructural effects [37].

ThermoCalc predicts the sigma phase instability at higher temperatures and is completely replaced by the B2#2 phase at 1000 °C. During in situ XRD, the sigma phase, which starts forming at 600 °C, retains its structure until 900 °C. This observation aligns with ThermoCalc predictions, though the start and finish temperatures of the phases show minor variations. ThermoCalc predictions are based on equilibrium phase diagrams, which assume that the material has sufficient time and conditions to reach a stable phase configuration [38]. In contrast, in situ XRD captures the real-time transformation of phases under experimental conditions, where the system may not reach equilibrium (more information in [39]) and the XRD capability to detect minor phase amounts (less than 5% threshold of total phase amounts) influences the observations.

Above 1000 °C, ThermoCalc predicts that the AlCrCoFeNi HEA consists mainly of BCC and B2#2 phases, with a minor amount of FCC phase. In situ XRD scans within this temperature range reveal the coexistence of FCC and B2 as the main phases. Upon cooling to room temperature, XRD confirms B2 and FCC as the main phases, differing from ThermoCalc predictions of B2 and BCC phases. Yeh et al. (2014) in study [40] reported the phase structure of the AlCrCoFeNi HEA when quenched and now comparing its behaviour during in situ XRD. The study observed that the alloy exhibited spinodal decomposition into A2 and B2 structures below 600 °C. Upon further heating to temperatures above 600 °C, the structure transitioned to a mix of FCC, sigma (σ), and B2 phases. At temperatures up to 960 °C, the sigma phase dissolved, resulting in a dual phase structure of FCC and B2.

The presence of multiple phases, including ordered and complex phases like the sigma phase, suggests that configurational entropy does not dominate phase stability across a wide temperature range. The multiple transformations and the presence of complex phases such as the sigma phase suggest that other thermodynamic factors, like enthalpy and Gibbs energy contributions from chemical ordering and phase transformations, play significant roles [41]. Thus, describing AlCoCrFeNi as a high entropy alloy is only truly valid just below the melting point where it remains a single-phase alloy. In this limited temperature range, the alloy is likely to have high configurational entropy typical of HEAs, which stabilises a simple solid solution phase [42]. Outside this range, the thermodynamics of the alloy favour the formation of multiple phases with lower configurational entropy, contradicting the typical HEA definition [43].

### 4.2. Oxidation Behaviour/Mechanism of AlCrCoFeNi HEA

**Figure 12:**
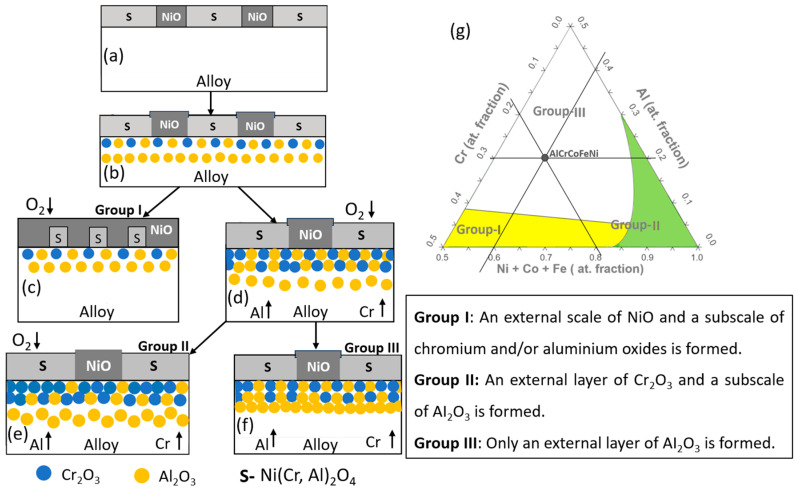
Shows composition-based oxidation mechanism schematic (**a**) rapid uptake of oxygen (**b**) Diffusion through the oxide into the alloy leads to the formation of Cr_2_O_3_ and Al_2_O_3_ subscales (**c**) In alloys with low chromium and aluminium concentrations, the subscale does not become continuous, and NiO predominates externally (Group I). (**d**) Alloys with higher concentrations of chromium and aluminium develop a continuous subscale, although aluminium is still oxidised internally beneath this duplex oxide layer. (**e**) For alloys with smaller aluminium concentrations, internal oxidation of aluminium continues, and the continuous duplex layer becomes enriched in chromium (Group II). (**f**) alloys with higher aluminium concentrations, the Al_2_O_3_ subscale zone becomes continuous beneath the duplex oxide layer (Group III). (**g**) Isothermal diagrams depict the compositional limits for these three oxidation mechanisms.

As shown in Figure 10b and Figure 11, the AlCrCoFeNi HEA effectively forms an Al_2_O_3_ layer, highlighting its suitability for environments requiring robust oxidation resistance. Alumina forming alloys are preferred due to Al_2_O_3_ extremely low deviation from stoichiometry, denoted as “δ”. Oxidation in metals and alloys is a high temperature phenomenon and follows a particular mechanism. Butler et al. [44] applied the Giggins–Pettit theory, originally developed for Ni-Cr-Al alloys [45], to elucidate the oxidation mechanisms of Al-Cr-Ni containing HEAs. This model categorizes the compositional space (i.e., Al, Cr and Co-Fe-Ni) into three different regimes as shown in Figure 12g is an empirically based structure that groups Ni-Cr-Al alloys into three distinct regimes based on their oxidation characteristics as shown in Figure 12:

Group-I includes alloys where the concentrations of Cr and Al are insufficient to form continuous Cr_2_O_3_ or Al_2_O_3_ scales. Instead, these alloys develop external scales composed of NiO, Ni_2_Cr_2_O_4_, and Ni_2_Al_2_O_4_ spinel phases, accompanied by internal oxidation of aluminium.

Group-II comprises alloys with sufficient chromium to enable the formation of a continuous external Cr_2_O_3_ scale, yet with aluminium concentrations low enough that only internal Al_2_O_3_ subscales are formed through internal oxidation.

Group-III includes alloys with high aluminium levels, fostering the selective oxidation of aluminium and leading to the formation of a continuous external Al_2_O_3_ scale.

Based on this, AlCrCoFeNi HEA in our study aligns with Group III of this model, characterised by high Al concentrations that promote the selective formation of an external Al_2_O_3_ scale. This categorization is corroborated by both the alloy’s behaviour in air and the compositional limits illustrated in Figure 12g, where the alloy’s position is marked by a black full circle. The alumina scale’s formation in AlCrCoFeNi HEA involved the outward diffusion of aluminium to react with surface oxygen, aligning with the Group III oxidation mechanism.

#### 4.2.1. Oxidation Behaviour in Low *p*(O_2_) Pressure

The oxidation behaviour of as-cast AlCrCoFeNi HEA was investigated in two different environments (i.e., vacuum, atmospheric air) during in situ XRD from room temperature to 1100 °C. In situ XRD spectra for vacuum conditions are provided in Figure 4a,b. During in situ XRD in vacuum, the formation of oxides was detected over 1000 °C. The lack of oxide phase detection at lower temperatures is primarily due to low *p*(O_2_) which significantly slows down the kinetics of oxidation and delayed the onset of oxide formation. Sato et al. (2002) [46] investigated the oxidation behaviours of modified stainless steel SUS316 (PNC316) and SUS316 at low *p*(O_2_) ranging from 10^−31^ to 10^−22^ atm at temperatures between 600 °C and 800 °C. They found that the oxidation rate constants were influenced by both *p*(O_2_) and temperature, with lower pressures resulting in slower oxidation kinetics. Specifically, they derived semi-empirical equations for the parabolic rate constants, showing lower rates constant values for lower *p*(O_2_) value. From this experience, we notice that a limitation in our current study is the lack of quantitative analysis through thermogravimetric analysis (TGA), which would provide valuable data on weight changes and oxidation rate constants to better understand the protective nature of these oxides. We plan to address this by exploring the integration of TGA into our existing setup in future work.

Considering that the data presented in Figure 10c of *p*(O_2_) oxidation and concentrations profiles of various elements show that aluminium oxide is found at the top and chromium is at alloy–oxide interface. This phenomenon is supported by the findings of Duval et al. (2010) [47], who reports that “the scale-growth mechanism was cationic for low (1 × 10^−5^ mbar) and medium *p*(O_2_) (0.2 mbar) conditions” where metal cations diffused outward to make oxide and promoted selective oxidation of alloying elements. Multiple evidence suggests that at low *p*(O_2_), the oxide develops through outward diffusion of cationic metallic species. SEM observations show nodular structures develop over a continuous oxide layer on the metal surface, indicating that Al^2+^ cations have diffused through the oxide layer. Additionally, EDX analysis (see Table in Figure 9) of the oxide surface shows that both Al and Cr have diffused and oxidised, with the oxide content increasing over time. The oxide layer structure shows chromium enrichment at interface (i.e., alloy-oxide), while the outer part primarily contains alumina. In this oxidation process, new oxide grows over the existing layer, which corresponds to an outward growth mechanism, and confirms that the growth occurs through diffusion of metal outwards to the surface.

The observed peaks in Figure 4 for Al_2_O_3_ and Fe_2_O_3_ at 1000 °C were not intense, indicating that the oxide layer formed was thin and not fully developed. This is consistent with the slow oxidation kinetics under low *p*(O_2_), where only a limited amount of oxide can form [48]. Further, EDX analysis of the oxide cross-section revealed a very thin oxide layer, less than 0.5 micro-meters in thickness. The absence of detectable Fe in the oxide layer but high counts of Cr and Fe at the interface suggests that Cr and Fe preferentially diffused from the bulk alloy. This phenomenon is closely related to the different diffusion rates of the different principal elements within multi component alloys. According to the conclusions of Tsai et al. [49] and Kai et al. [12], the elements order in terms of decreasing diffusion rate is as follows: Cr > Fe > Co > Ni. The EDX line analysis results are consistent with this differential diffusion phenomenon.

#### 4.2.2. Oxidation Behaviour in Air Conditions

XRD spectra during in situ XRD in air conditions suggested the formation of iron oxide (Fe_2_O_3_) and aluminium oxide (Al_2_O_3_) as the primary oxides on scan at 950 °C. The formation of ternary oxide CoCr_2_O_4_ was also detected at a temperature of 1100 °C [44]. During isothermal studies on oxidation behaviour of AlCrCoFeNi HEA its oxidation is reported at much lower temperatures. Fabrègue et al. [50] studied isothermal oxidation of AlCrCoFeNi HEA at different temperatures and reported the formation of Al_2_O_3_ even at a temperature of 800 °C, which suggests the oxidation process is both time- and temperature-dependent. During in situ XRD, the high heating rate and lower delay time at each scan temperature leads to oxide detection at higher temperature. The microstructure and oxide scale chemical composition of the surface formed under air condition were analysed using SEM/EDX. As shown in Figure 8, surface oxide is mostly from Al_2_O_3_ as the associated table of point analysis shows oxygen at% of 64 and aluminium of 32%, which shows that the surface oxide is exclusive from alumina. Figure 8b shows oxide scale and exposed surface (after spallation) chemical composition. The oxide is mainly aluminium while the exposed surface composition is mainly from Cr, Co, and Fe. This spallation probably happened due to thermal stresses and the mechanical mismatch between underlaying alloy and oxide layer. Significant reduction in aluminium content on the exposed surface confirms that aluminium is selectively oxidised, forming a protective Al_2_O_3_ layer on the surface.

A cross-sectional image of the AlCrCoFeNi HEA after oxidation during in situ XRD is presented in Figure 11. The thickness of the oxide formed is approximately 1.25 µm after completion of oxidation process. The clear distinction of double layer oxide is visible Al_2_O_3_ formed ~1.08 μm thick top layer while inner layer mostly from Cr is ~0.18 μm. The creation of double-layer oxides in AlCrCoFeNi HEAs is well-documented. According to Liu et al. (2019) [17], the Al-containing HEA’s oxidation behaviour shows a clear distinction between layers, with an outer Al_2_O_3_-rich layer and an inner Cr-rich layer. This structure arises due to the different oxidation rates and affinities of aluminium and chromium within the alloy, where aluminium tends to form a thicker oxide layer on the surface, while chromium accumulates underneath and contributes to the oxide inner layer. Additionally, research by Zhu et al. (2020) [51] on AlCoCrFeNi HEAs highlights that the oxide outer layer is mainly from Al_2_O_3_, while the inner oxide layer is enriched in Cr_2_O_3_, confirming the creation of oxide double-layer type structure during oxidation at elevated temperature.

The map concentration of elements also suggest that the scale is predominantly from Al_2_O_3_ followed by Fe Cr and Fe. The line scan analysis for the formed oxide shown in Figure 10a also shows that top oxide layer is from Al_2_O_3_ and minor oxide layer as visible is from chromia. The formation of this selection oxide layer agrees well with Giggins–Pettit theory [52] who divides alloys for high-temperature oxidation/corrosion into three groups based on scale composition, in other words, based on quantity of elements making protective oxides. Equi-atomic AlCrCoFeNi HEA falls in group-III capable selective oxidation and forming Al_2_O_3_ scale on the outer surface.

### 4.3. Interplay of Phase Stability and Oxidation in AlCrCoFeNi HEA

The as-cast AlCoCrFeNi alloy initially is an imperfect equilibrium state due to the cooling rate experienced after its melting. While upon heating, the alloy transitions from this unstable state, as evidenced by the in situ XRD analysis (Figure 4 and Figure 6). These changes were tracked progressively throughout from room temperature to 1100 °C in both, i.e., low *p*(O_2_) and air environments. The phase transitions and their transformation temperatures were recorded, along with the intensities of each phase, showing their relative amounts. In both low *p*(O_2_) and air environments, three phases are detected: FCC, BCC, and B2 in the substrate alloy. Additionally, the content of the FCC phase gradually increases with longer time and higher temperature, as indicated by the peak intensities, ultimately reaching a maximum at 1100 °C. Peaks for oxides such as Al_2_O_3_ and Fe_2_O_3_ are detected in vacuum, and Al_2_O_3_, Fe_2_O_3_, and CoCr_2_O_4_ are found in the air environment.

To illustrate the distribution of the three phases in the as-cast AlCoCrFeNi alloy after oxidation in vacuum and air environments at 1100 °C, the cross-sectional morphologies of polished specimens oxidised in these conditions are presented in Figure 9c,d. Initially, an Al-depletion layer with an FCC structure forms at the alloy surface due to the development of an Al_2_O_3_ scale. The thickness of this Al-depletion layer increases with increasing oxidation temperatures, correlating well with the increasing peak intensity of the FCC phase (Figure 4 and Figure 6). In air, the thickness of the FCC phase after Al-depletion is approximately 1.26 μm (Figure 9c and Figure 11), compared to less than 0.25 μm in a vacuum (Figure 9d). The transformation to FCC follows the depletion of Al from the substrate alloy, indicating an oxidation-dependent phase transformation in the AlCrCoFeNi HEA [27]. Additionally, a minor FCC phase precipitates along the grain boundaries within the bulk alloy (Figure 1d).

The virgin AlCoCrFeNi alloy primarily consists of periodic, interconnected, and nanostructured BCC and B2 phases (Figure 1b). Upon exposure to high-temperature oxidation, a significant transformation to the FCC phase occurs beneath the oxide layer, with minor transformations within the bulk alloy. Initially, the sizes of BCC and B2 phases in the virgin alloy sample are less than 200 nm (Figure 1b), increasing to about 500-700 nm after oxidation at 1100 °C (Figure 1d). The size of the FCC phase at grain boundaries grows from less than 300 nm (see inset in Figure 1a,b) to over 700 nm post-oxidation (Figure 1d). In the as-cast AlCoCrFeNi alloy, a nanostructured BCC and B2 spinodal decomposition mechanism is predominant [21]. Therefore, the nanostructured BCC and B2 phases remain stable at high temperatures (900–1100 °C) during the early stages of oxidation.

Thus, a triple-phase structure is clearly observed after oxidation in both air (with more FCC) and vacuum (with less FCC) environments (Figure 1b,d), which aligns with the XRD results shown in (Figure 4 and Figure 6). A continuous Al-depletion layer with an FCC structure forms beneath the Al_2_O_3_ scale due to the outward diffusion of Al to form Al_2_O_3_ (Figure 10a,c). Similar BCC to FCC phase transformations due to Al consumption are commonly observed in dual-phase commercial NiCoCrAlY-type alloys [53,54]. The bright contrast phase is identified as the FCC phase, while the dark contrast phase is the ordered B2 phase beneath the Al-depletion layer (Figure 10b). Notably, SEM-EDX point scans reveal a relatively low Ni content in the Al-depletion layer post-oxidation (see table in Figure 10). The original AlCoCrFeNi alloy comprises a NiAl-rich B2 phase and a NiAl-poor BCC phase (Figure 1b). The BCC phase with low Al content first transforms into the FCC structured Al-depletion layer due to Al_2_O_3_ scale formation. After oxidation at 1100 °C, the NiAl-rich B2 phase remains prominent, indicated by the brightest regions in the Ni maps, while the Al-depletion layer appears darker (Figure 11 elemental concentration maps). This observation is further confirmed by the SEM-EDX line scan analysis passing through both dark and bright regions in the bulk alloy and across the oxide layer and Al-depleted zone (Figure 10a,c).

Upon high-temperature oxidation, the AlCrCoFeNi high entropy alloy (HEA) undergoes a significant transformation from primarily BCC and B2 phases to an increased proportion of the FCC phase beneath the oxide layer. This phase transition is influenced by the oxidation environment, where a thicker FCC phase forms under air compared to vacuum due to thinner oxide formation and minimum aluminium depletion [55]. This transformation, evidenced by changes in phase proportions and morphological characteristics under varying conditions, illustrates the dynamic response of AlCrCoFeNi HEA to oxidation.

### 4.4. Predicted Oxides of AlCrCoFeNi HEA

**Figure 13:**
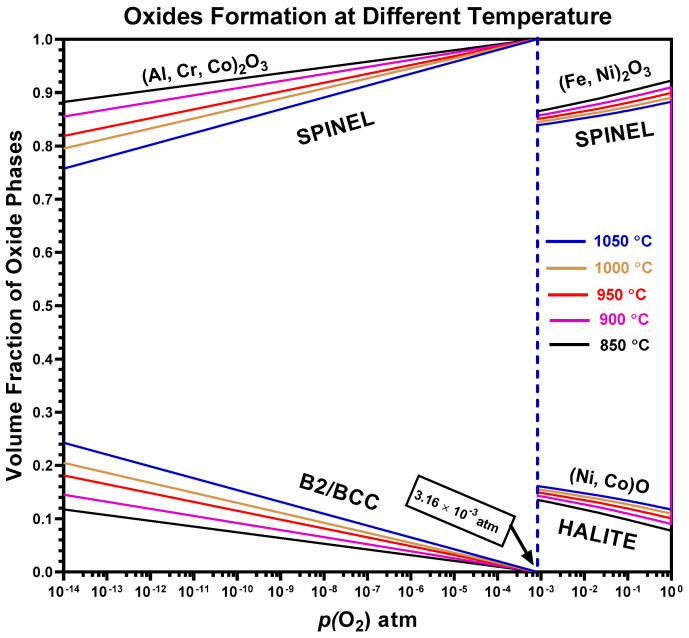
Thermodynamically stable phase oxide phase of AlCrCoFeNi HEA at different temperatures and varying oxygen partial pressure.

The ThermoCalc prediction map shown in Figure 13 for the AlCrCoFeNi high entropy alloy (HEA) gives a comprehensive understanding of how various oxide phases—halite and spinel—form under varying temperatures and oxygen partial pressures *p*(O_2_). At all temperatures, i.e., from 850 °C to 1050 °C, the alloy mainly forms spinel-type oxides. As shown in Figure 6, in situ XRD in air AlCrCoFeNi HEA formed CoCr_2_O_4_ spinel oxide as opposed to in low *p*(O_2_). Also, the findings of Hong et at (2019) support the high-volume prediction of spinel oxide type at high *p*(O_2_) as in their study [56] they found that in case of Fe magnetite (Fe_3_O_4_) is comparatively more stable than hematite (Fe_2_O_3_) provided there is adequate concentration of oxygen. But the trend of the graph at various temperature shows that as temperature increases the volume fraction of Halite type of oxide increases as its volume fraction at 850 °C and at 1.0 atm *p*(O_2_) is 0.077485 which becomes 0.117173 at 1050 °C. Similarly, the volume fraction of spinel oxides is 0.922515 at 850 °C and becomes 0.882827 at 1050 °C showing a downward trend with a corresponding uptrend for halite. Up till medium *p*(O_2_) the volume fraction of halite type of oxide increased from 0.117173 at 1 atm to 0.161182 at 8.0 × 10^−4^ atm while volume fraction of spinel at corresponding values decreased from 0.882827 to 0.838818. Shift in volume fraction of phases and phase structure noticed at *p*(O_2_) of 8.1 × 10^−4^ the volume fraction of halite phase transforms totally to spinel type of oxide while a new phase which is the alloy phase B2/BCC starts to grow from zero to higher values with decreasing *p*(O_2_). The study by Gao et al. (2002) investigated the influence of lower *p*(O_2_) 10^−15^–10^−22^ atm, on the oxidation behaviour of nickel- and iron-aluminides. At higher *p*(O_2_) 10^−20^ atm, nickel particles formed on Ni3Al surfaces, while Al oxidation occurred internally, and surface remained unoxidized. The finding of Gao et al. (2002) agreed well with the formation of B2/BBC phase predicted by ThermoCalc.

**Figure 14:**
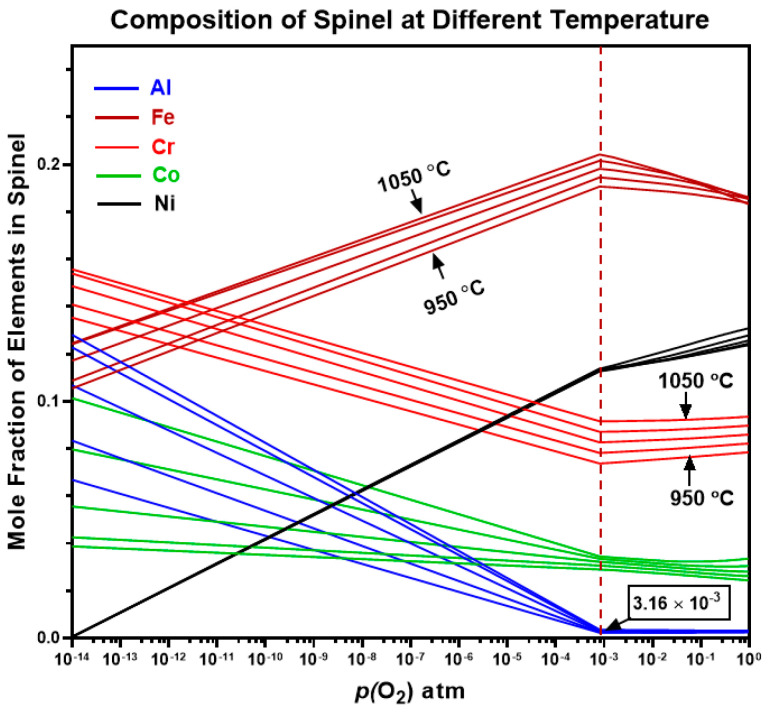
Elemental molar amount of ThermoCalc predicted stable spinel oxide phase for AlCrCoFeNi HEA.

The ThermoCalc predictions for the elemental composition of the spinel oxide phase in AlCrCoFeNi HEA reveal significant trends as shown in Figure 14. Iron (Fe) constitutes the largest fraction of the spinel oxide, with its proportion increasing from approximately 0.1 to 0.22 as temperature rises from 850 °C to 1050 °C and *p*(O_2_) decreases. At high *p*(O_2_), Nickel (Ni) is the second most significant element forming spinel oxide, increasing up to 0.17 at 1 × 10^−2^ atm.

At a critical value of *p*(O_2_) of 8.0 × 10^−4^ (atm), the composition of the spinel oxide changes significantly. Aluminium (Al) shows the highest gradient with changing *p*(O_2_), indicating a strong tendency to form spinel oxides, especially as temperature increases. Its fraction decreases from around 0.12 to almost zero as *p*(O_2_) increases. Chromium (Cr) is the second most significant contributor to the spinel phase at lower *p*(O_2_), with its mole fraction increasing from 0.08 to 0.16 as *p*(O_2_) decreases, and higher temperatures, followed by Cobalt (Co), which also shows a trend of increased spinel formation with decreasing *p*(O_2_).

Overall, Fe and Ni dominate the spinel formation at high *p*(O_2_), while Fe, Cr, and Al are more prevalent spinel formers at lower *p*(O_2_). This demonstrates the complex interaction between temperature, *p*(O_2_), and the resulting oxide composition in AlCrCoFeNi HEA.

**Figure 15:**
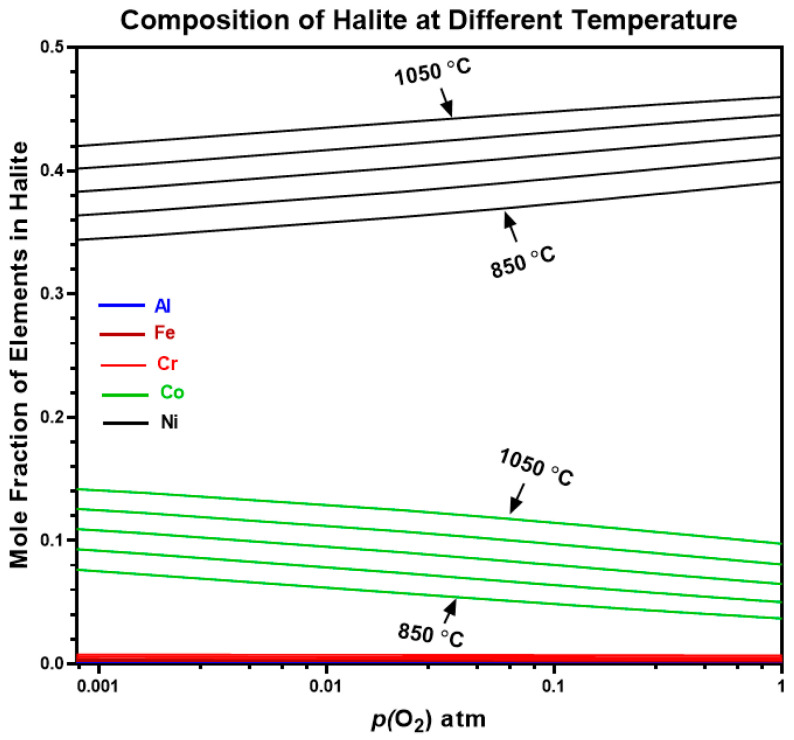
Shows mole fraction of elements in halite type of oxide phase for AlCrCoFeNi HEA at different temperature.

The ThermoCalc predictions for the elemental composition of the halite oxide phase in AlCrCoFeNi HEA are shown in Figure 15. Nickel (Ni) is the predominant element forming halite-type oxides, constituting up to 90% of the halite phase at high *p*(O_2_), with its proportion increasing further with rising temperatures. Cobalt (Co) is the second most significant contributor to halite oxides at lower *p*(O_2_). However, the formation of Ni halite decreases with decreasing *p*(O_2_) while Co rises with lowering the oxygen content. Aluminium (Al), Chromium (Cr), and Iron (Fe) have near-zero mole fractions at high *p*(O_2_). ThermoCalc does not predict any element forming halite at oxygen pressure lower than 10^−3^ atm. However, the primary phase (B2/BCC) of alloy is predicted along with spinel oxide at lower pressure values.

## 5. Conclusions

The AlCrCoFeNi phase stability and oxidation were systematically investigated using in situ HTXRD and ThermoCalc simulations. This study advances the understanding of the high-temperature behaviour of AlCrCoFeNi HEA, providing a foundation for future research on contribution of phases that promote the formation of continuous alumina/chromia oxide layer for development AlCrCoFeNi HEA as a coating material for high-performance and longevity in extreme environments.

Key findings from this study include:

- The alloy exhibits B2, BCC, Sigma and FCC, phases at various temperatures, with notable phase transitions occurring around 600 °C and 1000 °C. At intermediate temperatures, sigma phase is stable but decomposes at higher temperatures and lower temperatures as predicted by ThermoCalc.
- The creation of oxide layers of protective nature, primarily Al_2_O_3_ and Cr_2_O_3_, was observed, contributing to the alloy’s oxidation resistance. The oxide layer structure was characterised by a thick alumina top layer and a thinner chromia sublayer.
- During in situ HTXRD, the fine maze structured microstructure transformed into ultrafine micro-platelet microstructure with significant formation of FCC at expense of BCC/B2 phase. Also, it was found that thick oxide formation results in a thick layer of Al-deleted FCC phase under the oxide layer.
- Thermodynamic simulations provided valuable insights into the phase evolution and were confirmed experimentally with minor variations in temperature for different phases. Predicted spinel-type oxides as predominant oxide at high *p*(O_2_), corresponds to experimentally spinel oxide.
- The in situ HTXRD allowed real-time monitoring of phase transformations and oxide formation, highlighting the dynamic nature of the oxidation process and the significant influence of *p*(O_2_) on the oxidation behaviour.

## Figures and Tables

**Table 1:** List of variables for initialization of ThermoCalc calculations.

Temperature	1000 (°C)	
Pressure	101,325 (Pa)	
System Size	1.0 (Mole)	
Composition (at.%)	Al	20.0
	Ni	20.0
	Co	20.0
	Fe	20.0
	Cr	20.0
	O	05.0
O_2_ Activity	Min	1.0 × 10^−14^
	Max	1.0
	Steps	100

**Table 2:** Chemical composition of AlCrCoFeNi HEA from EDX chemical analysis.

Composition (at%)	Co	Cr	Al	Fe	Ni
Nominal	20.0	20.0	20.0	20.0	20.0
Measured (EDS)	20.72 ± 0.2	20.95 ± 0.2	17.26 ± 0.1	20.72 ± 0.2	20.35 ± 0.2

## Data Availability

The original contributions presented in the study are included in the article, further inquiries can be directed to the corresponding author.
